# Emergency Laparoscopic Repair of an Iatrogenic Gastric Perforation in a Hiatal Hernia following a Failed Endoscopic Closure

**DOI:** 10.1155/2020/5060962

**Published:** 2020-03-25

**Authors:** Benjamin L. Reed, Lawrence E. Tabone, Nova Szoka, Salim Abunnaja

**Affiliations:** Department of Surgery, West Virginia University School of Medicine, One Medical Center Drive, PO Box 9238, Morgantown, WV 26508, USA

## Abstract

Iatrogenic gastrointestinal perforation is a rare, life-threatening complication of endoscopic procedures, which requires either endoscopic or surgical repair. We report the account of an 82-year-old woman with an iatrogenic gastric perforation of a hiatal hernia secondary to an endoscopic retrograde cholangiopancreatography (ERCP) procedure. Despite immediate recognition of the complication and endoscopic closure with through-the-scope (TTS) clips, the patient developed mediastinitis, peritonitis, and sepsis. She subsequently underwent an emergency laparoscopic hiatal hernia dissection and repair of the perforation with mediastinal and peritoneal washout. Given the patient's age and the degree of insult, subdiaphragmatic anchoring with abdominal drain placement was performed, and the hiatus was left open for additional drainage. The use of a side-viewing duodenoscope with the presence of a large hiatal hernia contributed to the risk of gastric perforation. We conclude that performing endoscopic procedures in patients with a known hiatal hernia should be carefully undertaken. If a perforation in such patients occurs, laparoscopic repair of such complications is feasible as demonstrated in this case video.

## 1. Introduction

Endoscopy is a safe and effective treatment modality for a variety of gastrointestinal disorders, with a complication rate of <1% and a mortality of <0.01% [1]. Among the possible complications of endoscopy is visceral perforation or tear along the surveilled portion of the gastrointestinal tract, which may occur due to overinsufflation or puncture from the endoscope or endoscopic instruments. Not all endoscopies are equal with regard to perforation risk, with some patients carrying a much higher chance of suffering this complication. Such patients are typically older, are admitted to a hospital, and have comorbidities such as active inflammatory bowel disease, COPD, malignancy, or concomitant steroid use. Additionally, small bowel enteroscopy places patients at a higher risk of perforation [2, 3].

Repair of full-thickness perforations is necessary to prevent contamination of the peritoneal cavity with subsequent peritonitis and sepsis. Such repair has traditionally been operative, though procedural management with through-the-scope (TTS) clipping has recently come to the fore as a viable, proven option [4]. In addition to definitive closure of the defect, patients must be serially surveilled, with a low threshold to pursue additional salvage operations. Other considerations for management should include a switch to carbon dioxide insufflation and decompression of pneumoperitoneum [5]. In this report, we present the case of a patient who suffered a gastric perforation during endoscopic retrograde cholangiopancreatography (ERCP). Initial management with TTS clipping failed in this instance, requiring operative intervention.

## 2. Case Report

An 82-year-old woman presented as a transfer from an outside facility with painless, obstructive jaundice. She observed yellowing of her skin, poor appetite, and fatigue for two days, as well as dark brown urine. She was otherwise healthy and independent at home. She did not smoke and had no family history of malignancy.

Diagnostic workup at the outside facility was notable for elevated total bilirubin to 14.4 mg/dL, alkaline phosphatase to 1,004 U/L, and white blood cell count was elevated at 23.6 × 10^3^/*μ*L. Magnetic resonance cholangiopancreatography (MRCP) had shown intra- and extrahepatic biliary dilation with evidence of a stricture in the distal common bile duct (CBD) suggestive of a pancreatic head mass. It also revealed the presence of a large, type III hiatal hernia, which had been previously asymptomatic other than mild reflux disease.

Given the concern for cholangitis, the patient was taken for an endoscopic ultrasound with subsequent ERCP to further evaluate her pathology and relieve her biliary obstruction. Endoscopy confirmed the presence of a large, type III hiatal hernia, which made advancing the endoscope into the duodenum difficult. The endoscopists were still able to proceed with ultrasound and were successful in identifying and obtaining biopsies of a mass in the pancreatic head. However, ERCP was aborted as the scope could not be advanced past the second portion of the duodenum. As the scope was being removed from the stomach, a small, linear tear was noted in the stomach mucosa along the greater curvature of the fundus, though the endoscopist believed it to be a partial-thickness tear. This was closed with serially placed 5 mm endoscopic clips, and complete approximation of the mucosa was seen.

**Figure 1:**
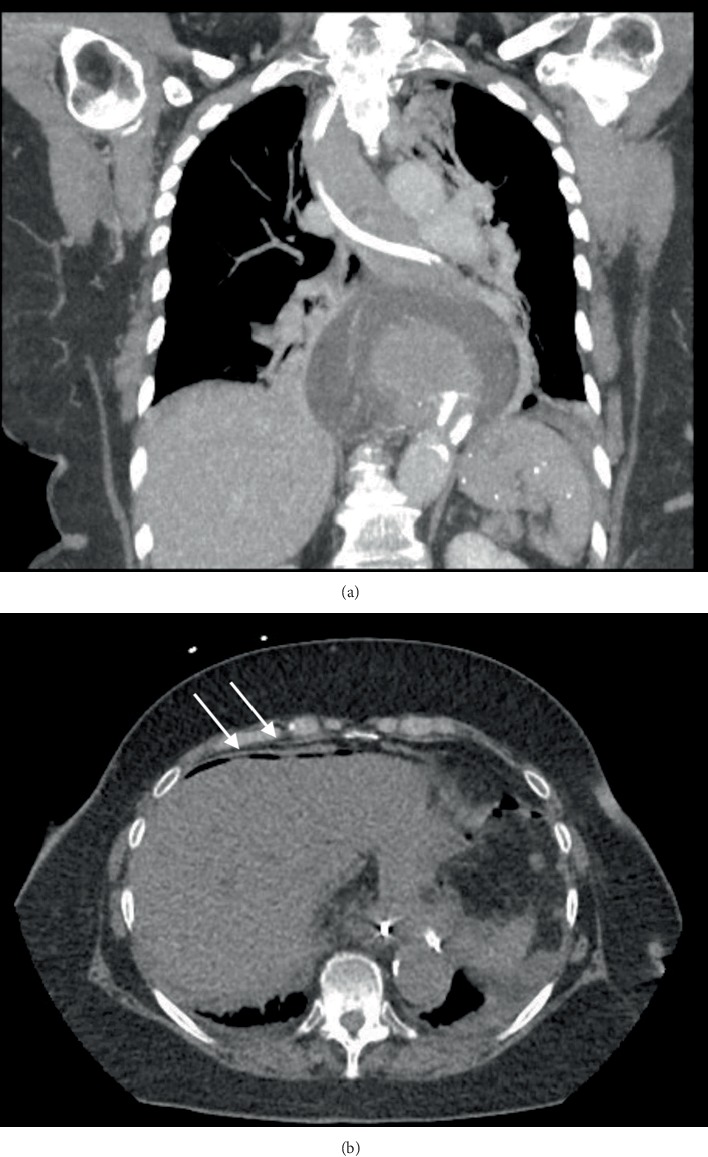
CT scan showing intra-abdominal and mediastinal free air and extraluminal contrast from the patient's gastric perforation. (a) Coronal view. (b) Axial view.

**Figure 2:**
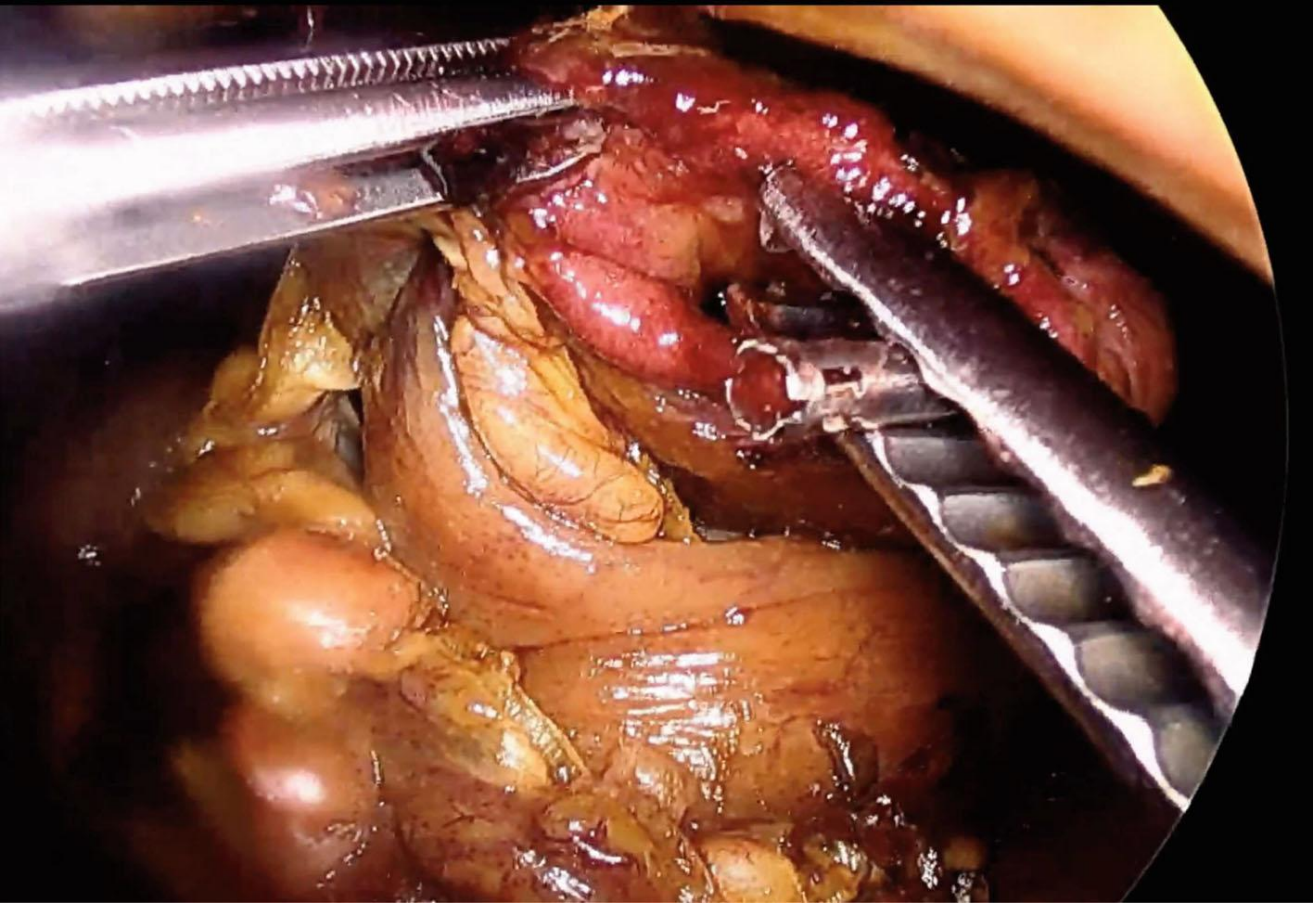
A still from the intraoperative video showing the perforation along the greater curvature of the fundus.

The patient was subsequently placed on IV antibiotics and closely observed. Unfortunately, she progressed to develop signs of mediastinitis, peritonitis, and sepsis. A subsequent computed tomography (CT) scan showed a persistent hernia with intra-abdominal and mediastinal free air and extraluminal contrast (Figures 1(a) and 1(b)). She was then taken to the operating room emergently for laparoscopic exploration (see Supplemental Video with narration (available here)). She was found to have gross bilious peritonitis and mediastinitis. The patient underwent a laparoscopic dissection and reduction of her hiatal hernia with the repair of her gastric perforation and abdominal and mediastinal washout (Figure 2). The perforation along the greater curvature of the fundus was repaired in layers with PDS suture. Given the high-acuity presentation and patient age, reduction of the hiatal hernia and gastropexy were performed. Subdiaphragmatic anchoring was chosen instead of the closure of the hiatus to allow for continued drainage of the mediastinal cavity. This was accomplished using a gastrostomy tube and full abdominal wall thickness gastropexy sutures. The gastropexy approximated the anterior gastric wall at the site of the gastrostomy to the upper abdominal wall to provide traction on the gastric fundus to prevent reincarceration through the hiatus. A mediastinal drain was placed. Before recovering from anesthesia, she underwent placement of a cholecystostomy tube and percutaneous transhepatic catheter for biliary drainage and biliary decompression.

The patient had a slow but steady recovery following this operation. Pathologic examination of the fine-needle biopsy of her pancreatic head mass showed atypical cells. She was discharged to a rehabilitation facility on postoperative day 12. At that time, she was able to tolerate a regular diet with the gastrostomy tube clamped and biliary drains internalized. She eventually transferred care to a facility that was closer to her family, where a palliative bypass procedure was performed due to locally invasive disease to the portal and superior mesenteric veins. Intraoperative frozen section confirmed adenocarcinoma.

## 3. Discussion

Iatrogenic perforation during endoscopy has an incidence of about 0.001% in retrospective reviews, with injuries occurring throughout the GI tract. There are both physiologic and anatomic risk factors for perforation. Physiologic risk factors include markers for systemic inflammatory states such as active inflammatory bowel disease, COPD, steroid use, increased age, or inpatient hospital admission. Anatomic risk factors include diverticula, malignancy, or small bowel enteroscopy [2, 3]. With upper endoscopy in particular, roughly half of perforations occur in the esophagus, frequently associated with dilatory procedures. The next most common site of perforation is the duodenum, with far fewer perforations occurring in the stomach, jejunum, and bile ducts [6]. In addition to the risk factors already discussed, risk for perforation on upper endoscopy can be increased in the presence of anatomic anomalies of the upper GI tract, such as anterior cervical osteophytes, Zenker's diverticula, esophageal strictures, and malignancies [1]. This particular injury was attributable to the patient's large, hiatal hernia, as well as the use of the side-view endoscope necessary for ERCP.

Though perforations from endoscopy may be managed conservatively, this injury pattern has a mortality that approaches 20% and thus warrants diligent surveillance [1]. When encountering a perforation, the endoscopist should be sure to document the size and location of the tear, whether endoscopic treatment was feasible and/or attempted, and whether carbon dioxide or air was used for insufflation. Nonoperative management is possible in select patients. In one series of 77 patients with perforation secondary to upper endoscopy, nonoperative management was attempted in half, though the failure rate for nonoperative management was 18% (TTS clips were not attempted) [6]. Esophageal injuries in this series were more likely to be able to accomplish nonoperative management, perhaps because of the availability of endoscopically placed stents to occlude perforations. In perforations that are not amenable to stenting, treatment by TTS clips has been shown to be safe and effective, decreasing the failure rate of nonoperative management to as low as 5% [7, 8]. Alternative endoscopic approaches to the closure of perforations have included endoscopic suture placement with Apollo Endostitch or the use of covered stents, as mentioned above. TTS clip placement likely failed in this case because the tear was in the herniated portion of the stomach and subjected to increased gastric pressure from distal obstruction at the crura.

This case also demonstrates the feasibility of laparoscopic repair for iatrogenic gastric perforation, even in cases where the repairs are anatomically complex and the patient's disease is extensive. Though TTS clipping failed in this case, such closures should be attempted in all cases of iatrogenic endoscopic perforation where the anatomy is favorable. Subdiaphragmatic anchoring with gastrostomy, gastropexy, and drainage was an effective and safe approach in this case, as closure of the hiatus would have risked having undrained contamination in the mediastinum. Laparoscopic repairs of this complexity require familiarity with minimally invasive equipment and techniques. In the absence of specialty care, an open approach may be more appropriate.

## Supplementary Materials

Supplementary Materials Supplemental Video: narrated video of the surgical intervention. The patient underwent a laparoscopic dissection and reduction of her hiatal hernia with repair of her gastric perforation and abdominal and mediastinal washout.
